# Gene therapy with the TRF1 telomere gene rescues decreased TRF1 levels with aging and prolongs mouse health span

**DOI:** 10.1111/acel.12677

**Published:** 2017-09-24

**Authors:** Aksinya Derevyanko, Kurt Whittemore, Ralph P. Schneider, Verónica Jiménez, Fàtima Bosch, Maria A. Blasco

**Affiliations:** ^1^ Telomeres and Telomerase Group Molecular Oncology Program Spanish National Cancer Centre (CNIO) Melchor Fernández Almagro 3 Madrid E‐28029 Spain; ^2^ Center of Animal Biotechnology and Gene Therapy Department of Biochemistry and Molecular Biology School of Veterinary Medicine Universitat Autònoma de Barcelona Bellaterra 08193 Spain; ^3^ Centro de Investigación Biomédica en Red de Diabetes y Enfermedades Metabólicas Asociadas (CIBERDEM) Madrid Spain

**Keywords:** adeno‐associated serotype 9 vector, aging, shelterin, telomeres, Trf1

## Abstract

The shelterin complex protects telomeres by preventing them from being degraded and recognized as double‐strand DNA breaks. TRF1 is an essential component of shelterin, with important roles in telomere protection and telomere replication. We previously showed that TRF1 deficiency in the context of different mouse tissues leads to loss of tissue homeostasis owing to impaired stem cell function. Here, we show that TRF1 levels decrease during organismal aging both in mice and in humans. We further show that increasing TRF1 expression in both adult (1‐year‐old) and old (2‐year‐old) mice using gene therapy can delay age‐associated pathologies. To this end, we used the nonintegrative adeno‐associated serotype 9 vector (AAV9), which transduces the majority of mouse tissues allowing for moderate and transient TRF1 overexpression. AAV9‐TRF1 gene therapy significantly prevented age‐related decline in neuromuscular function, glucose tolerance, cognitive function, maintenance of subcutaneous fat, and chronic anemia. Interestingly, although AAV9‐TRF1 treatment did not significantly affect median telomere length, we found a lower abundance of short telomeres and of telomere‐associated DNA damage in some tissues. Together, these findings suggest that rescuing naturally decreased TRF1 levels during mouse aging using AAV9‐TRF1 gene therapy results in an improved mouse health span.

## Introduction

Aging is characterized by a time‐dependent functional impairment of the organism in which several molecular pathways have been demonstrated to play a causal role (reviewed in Gladyshev, 2016; López‐Otín *et al*., 2013). Current efforts are directed at specifically targeting these fundamental aging events because intervening in these molecular pathways could delay or prevent age‐related diseases and increase lifespan (reviewed in López‐Otín *et al*., 2013). Telomere shortening has been identified as one of the primary hallmarks of aging (Blasco, 2005; López‐Otín *et al*., 2013; Martínez & Blasco, 2017).

Mammalian telomeres are heterochromatic structures at the end of linear chromosomes that consist of TTAGGG repeats bound by an array of associated proteins known as shelterin, which prevent chromosome ends from being recognized as double‐strand DNA breaks and from chromosome end‐to‐end fusions (reviewed in de Lange, 2005; Blackburn, 2000). Telomeres evolved in eukaryotic cells as a solution to the ‘end‐replication problem’, which refers to the incomplete DNA replication of the ends of linear chromosomes by the DNA replication machinery (Watson, 1972; Olovnikov, 1973), thus leading to telomere shortening with each round of cell division (Harley *et al*., 1990). When telomeres shorten below a threshold length, they become dysfunctional and this activates a DNA damage response (DDR) at chromosome ends, which in turn leads to the activation of apoptotic and/or cellular senescence programs and thus to the deregulation of many functional processes (Blackburn, 2000; Martens *et al*., 2000; O'Sullivan & Karlseder, 2010).

Telomerase is a reverse transcriptase (TERT) that elongates telomeres *de novo* by adding telomeric repeats on chromosome ends using as template an RNA component (TERC), thus preventing telomere erosion (Greider & Blackburn, 1985). However, mammalian cells stop expressing telomerase in the majority of tissues after birth (Blasco *et al*., 1995; Schaetzlein *et al*., 2004), leading to progressive telomere erosion throughout the lifespan of the organism. Telomere shortening has been demonstrated to be sufficient to trigger age‐related pathologies and shorten lifespan in mice (Blasco *et al*., 1997; Lee *et al*., 1998; Blasco, 2005). Similarly, humans suffering from the so‐called telomere syndromes, characterized by mutations in telomerase and other telomere maintenance genes, also show premature age‐related pathologies (Gilson & Londoño‐Vallejo, 2007; Armanios & Blackburn, 2012).

Telomerase reactivation has been envisioned as an strategy to maintain telomeres and therefore to increase the proliferative potential of tissues, both in the telomere syndromes and in age‐related conditions. We first demonstrated that constitutive TERT expression in the context of cancer‐resistant mice was sufficient to maintain longer telomeres and less DNA damage with aging, as well as to delay age‐related pathologies and increase mouse longevity by 40% (Tomás‐Loba *et al*., 2008). More recently, telomerase overexpression in adult and old mice using nonintegrative gene therapy vectors was sufficient to delay physiological aging and increase both median and maximum lifespan in wild‐type mice in the absence of increased cancer (Bernardes de Jesus *et al*., 2012). Telomerase reactivation in mice with critically short telomeres owing to telomerase deficiency was also able to reverse tissue degeneration (Jaskelioff *et al*., 2011). Finally, small molecule telomerase activators have been also described to delay some features of aging (Bernardes de Jesus *et al*., 2011; Harley *et al*., 2011).

In addition to telomerase, the shelterin complex is also critical for the protection of telomeres. Shelterin consists of six proteins, namely TRF1, TRF2, POT1, TIN2, TPP1, and RAP1 (reviewed in de Lange, 2005). TRF1 is one of the key components of the shelterin complex (Bianchi *et al*., 1999). Through its interaction with TIN2, TRF1 binds to TRF2, playing an important role in shelterin complex assembly (Xin *et al*., 2008; Diotti & Loayza, 2011). TRF1 is important both to prevent telomere fusions as well as for the replication of telomeric regions (Sfeir *et al*., 2009). In particular, deletion of TRF1 in mouse embryonic fibroblasts (MEFs) results in induction of senescence, as well as in chromosome fusions and multitelomeric signals (aberrant number of telomeric signals per chromosome end) (Martínez *et al*., 2009; Sfeir *et al*., 2009). Importantly, these effects of TRF1 abrogation are independent of telomere length, as TRF1 deletion uncaps telomeres independently of telomerase and cell division (Karlseder *et al*., 2003; Martínez *et al*., 2009; Sfeir *et al*., 2009). In addition, conditional TRF1 abrogation in various mouse tissues has demonstrated the importance of TRF1 for tissue regeneration and tissue homeostasis (Martínez *et al*., 2009; Beier *et al*., 2012; Schneider *et al*., 2013; Povedano *et al*., 2015). Indeed, high TRF1 levels mark stem cell compartments as well as pluripotent stem cells and are essential to induce and maintain pluripotency (Boué *et al*. 2010; Schneider *et al*., 2013). In this regard, we previously showed that TRF1 is a direct transcriptional target of the pluripotency factor Oct3/4 (Schneider *et al*., 2013).

Intriguingly, TRF1 levels have been shown to decrease with cell passaging *in vitro* and subsequent overexpression of TRF1 in these cells reduced DNA damage at telomeres and decreased senescence, suggesting that decreased TRF1 levels with cell passaging can contribute to senescence *in vitro* (Hohensinner *et al*., 2016).

Given the importance of TRF1 for organismal viability and tissue homeostasis, here we set to address whether TRF1 levels vary with aging *in vivo* both in mouse and human tissues, as well as to study the potential therapeutic effects of TRF1 increased expression in delaying aging‐associated pathologies *in vivo*. A previous work of our group showed that constitutive TRF1 overexpression acted as a negative regulator of telomere length, mediating telomere cleavage by XPF nuclease. To circumvent this undesired effect of TRF1 overexpression, here we induced moderate and transient TRF1 overexpression in adult (1 year of age) and old (2 years of age) mice using nonintegrative adeno‐associated gene therapy vectors (AAV) that can transduce many different tissues but their expression is diluted as cells proliferate.

The results shown here demonstrate that TRF1 levels decrease with age both in mice and in humans. Furthermore, we demonstrate that transient TRF1 expression through the use of AAV9‐TRF1 gene therapy in wild‐type mice is able to improve mouse physiological health span as indicated by improvements in different markers of aging.

## Results

### TRF1 levels decrease with increasing age both in mice and in humans

**Figure 1:**
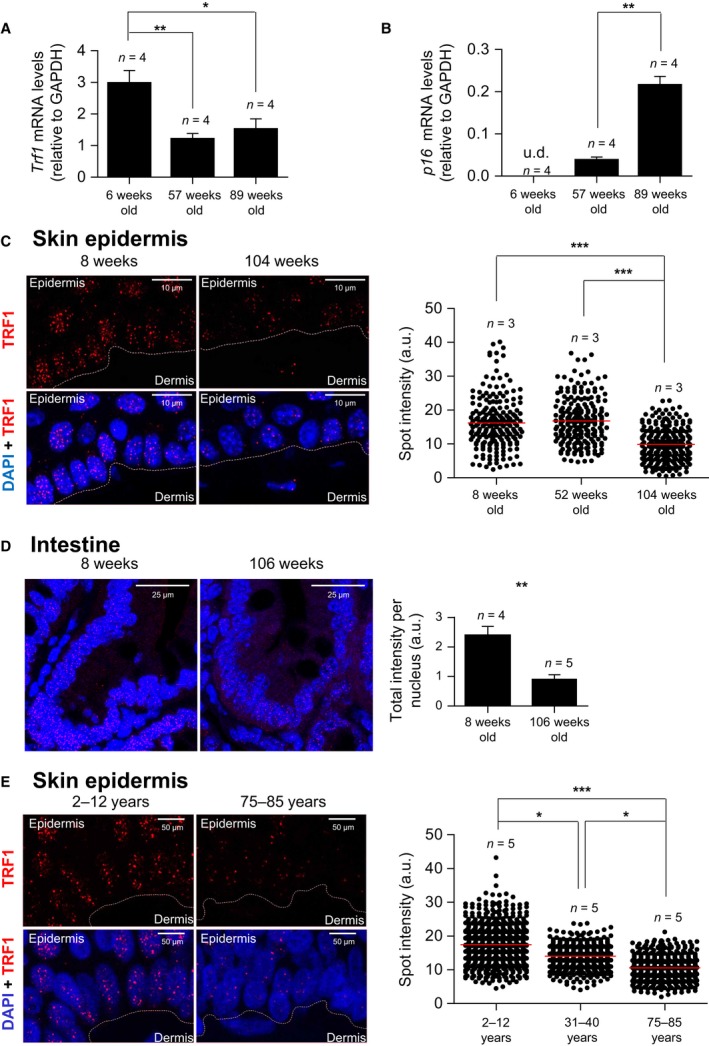
Decrease in TRF1 levels with aging in mice and humans. (A,B) *Trf1* (A) and *p16* (B) mRNA levels determined by RT–qPCR in tail skin epidermis from 6‐, 57‐, and 89‐week‐old mice. (C) Representative images of TRF1 (in red) and DAPI (in blue) and quantification of TRF1 protein levels measured by immunofluorescence analysis in mice of 8, 52, and 104 weeks old in back skin epidermis. (D) Representative images of TRF1 (in red) and DAPI (in blue) and quantification for TRF1 immunofluorescence analysis in mice of 8 weeks and 106 weeks old in intestine. (E) Representative images of TRF1 (in red) and DAPI (in blue) and quantification of TRF1 protein levels measured by immunofluorescence analysis in skin epidermis of young (2–12 years), middle‐age (31–40 years), and old (75–85) humans. *Trf1* and *p16* mRNA values are normalized to GAPDH. Student's *t*‐test was used for statistical analysis. Error bars represent SEM. U.d.—undetermined value. *P <* *0.05; **P <* *0.01; ***P <* *0.001. *n* indicates the number of mice/individuals. For each experiment, images were acquired with the same resolution and exposure parameters.

To address whether the decrease in TRF1 is associated with physiological aging *in vivo*, here we determined both mRNA and protein TRF1 levels in the epidermis of wild‐type mice at different ages including young (6‐ to ‐8 weeks old), adult (52‐ to ‐57 weeks old), and old mice (89‐ to ‐104 weeks old). We found significantly decreased *Trf1* mRNA levels in the epidermis of adult and old wild‐type mice compared to young mice (Fig. 1A). As an independent molecular marker of aging, we also measured p16 (a mediator of cellular senescence) mRNA levels, which are known to increase with age in almost all tissues in rodents and humans (Krishnamurthy *et al*., 2004; Satyanarayana & Rudolph, 2004). As expected, p16 mRNA levels were higher in the epidermis of middle‐aged mice compared to young mice, and this was further increased in old mice (Fig. 1B).

We also found decreased TRF1 protein expression levels in the mouse epidermis at different ages. To this end, we used immunofluorescence with antibodies against the TRF1 protein. TRF1 fluorescence was significantly decreased in old mice compared to both adult and young mice (Fig. 1C). We extended these findings to the intestine, where we also observed a significant decrease in TRF1 protein levels in the old mice group compared to young mice group (Fig. 1D).

Importantly, we also found decreased TRF1 levels with aging in the human epidermis. In particular, we performed TRF1 immunofluorescence on human skin samples from young (2‐ to ‐12 years old), adult (31‐ to ‐40 years old), and old (75‐ to ‐85 years old) individuals and observed significantly decreased TRF1 levels with age. In particular, adult human skin showed significantly lower TRF1 levels compared to young skin, and TRF1 levels were further decreased in old skin samples (Fig. 1E).

In the case of postmitotic tissues, we saw decreased TRF1 expression with aging in the muscle tissue in mice (Fig. S1A), but not in the liver (Fig. S1B).

In summary, these findings indicate that TRF1 levels decrease with age in mice and humans, at least in the majority of tissues that have been studied here.

### AAV9‐TRF1 treatment increases TRF1 levels in multiple mouse tissues

As TRF1 levels decrease with aging in mice and humans, we next set to study whether we could rescue phenotypes associated with aging *in vivo* by increasing TRF1 expression in adult and old mice. As we previously described that constitutive TRF1 overexpression in transgenic mice leads to XPF‐dependent telomere shortening (Muñoz *et al*., 2009), here we set to overexpress TRF1 in a moderate and transient manner. To this end, we used recombinant AAV vectors, which are nonintegrative (Ayuso *et al*., 2010), thus leading to a transient TRF1 overexpression. In particular, we used the AAV9 serotype, also previously used by us to deliver the TERT telomerase gene to many different adult tissues in mice (Bernardes de Jesus *et al*., 2012; Bär *et al*., 2014; Bar *et al*., 2016). AAV9 has a number of attractive properties such as poor immunogenicity, high transduction efficiency in a wide range of tissues, and the ability to cross the blood–brain barrier (Ayuso *et al*., 2010).

**Figure 2:**
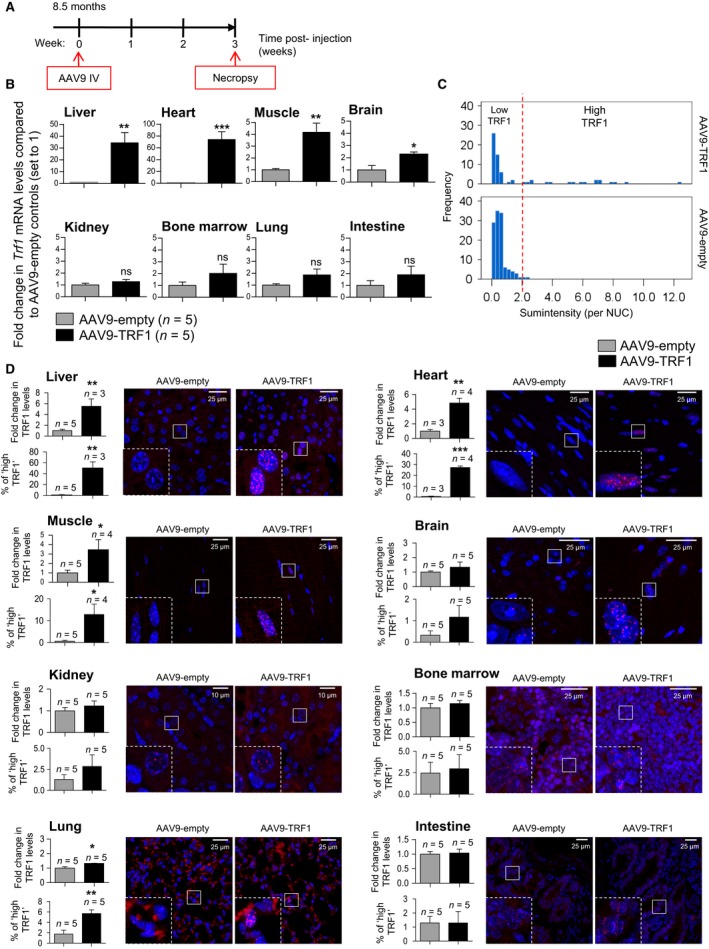
AAV9‐TRF1 transduction efficiency. (A) Scheme of mice injection for the transduction efficiency study. (B) *Trf1* mRNA levels determined by RT–qPCR in several murine tissues injected with AAV9‐TRF1 vector compared to mice injected with AAV9‐empty vector (set to 1). Five mice per group were used. *Trf1* mRNA values are normalized to GAPDH. (C) Histogram of the distribution of nuclei by their sum intensities calculated in muscle tissue of mice injected with AAV9‐TRF1 and AAV9‐empty. Nuclei with ‘High TRF1’ levels are determined as nuclei with intensities above the 99th percentile of the control group. (D) Representative images of TRF1 (in red) and DAPI (in blue) and quantification for TRF1 protein level measured by immunofluorescence analysis in various murine tissues in mice injected with AAV9‐TRF1 vector compared to AAV9‐empty vector (set to 1). Amplified images are shown in the insets. Student's *t*‐test was used for statistical analysis. Error bars represent SEM. *P <* *0.05; **P <* *0.01; ***P <* *0.001. *n* indicates a number of mice. Within each tissue, images were acquired with the same resolution and exposure parameters.

First, to assess the efficiency of transduction and expression of the AAV9‐TRF1 vector, we intravenously injected 8.5‐month‐old mice with AAV9‐TRF1 and 3 weeks postinjection determined TRF1 mRNA and protein levels in various tissues (Fig. 2A,B,D). Upon AAV9‐TRF1 transduction, we found significantly upregulated TRF1 mRNA levels in the liver, heart, muscle, and brain (Fig. 2B). However, we did not see a significant increase of the rest of the shelterin components (Fig. S2). We also found increased TRF1 mRNA levels in kidney, bone marrow, lung, and intestine, although in these cases, the differences did not reach significance (Fig. 2B). Using immunofluorescence analysis with anti‐TRF1 antibodies, we confirmed TRF1 protein overexpression both when measuring mean TRF1 fluorescence levels and the percentage of nuclei with ‘high TRF1’ levels. In particular, cells were designated as ‘high TRF1’ if the TRF1 levels were above the 99th percentile determined from the control group (Fig. 2C). We found increased TRF1 protein levels in liver, heart, muscle, and lung, when using both parameters (Fig. 2D). This pattern of overexpression of TRF1 using AAV9 is consistent with previous reports (Zincarelli *et al*., 2008; Bernardes de Jesus *et al*., 2012; Schuster *et al*., 2014). Thus, using AAV9‐TRF1 vectors, we can efficiently deliver TRF1 to different adult tissue types in the mouse.

### AAV9‐TRF1 treatment delays physiological mouse aging

To study whether AAV9‐TRF1 treatment was able to delay aging and age‐related phenotypes in mice, middle‐aged (1 year old) and old (2 year old) mice were intravenously injected with a single dose of either AAV9‐TRF1 or AAV9‐empty vectors. Upon treatment with the vectors, the mouse cohorts were longitudinally followed to determine different parameters of aging, cancer, as well as overall survival. Telomere length in blood samples was also determined longitudinally as a molecular biomarker of aging. At the end point of the experiments, a full histopathological analysis was performed.

#### Neuromuscular coordination

**Figure 3:**
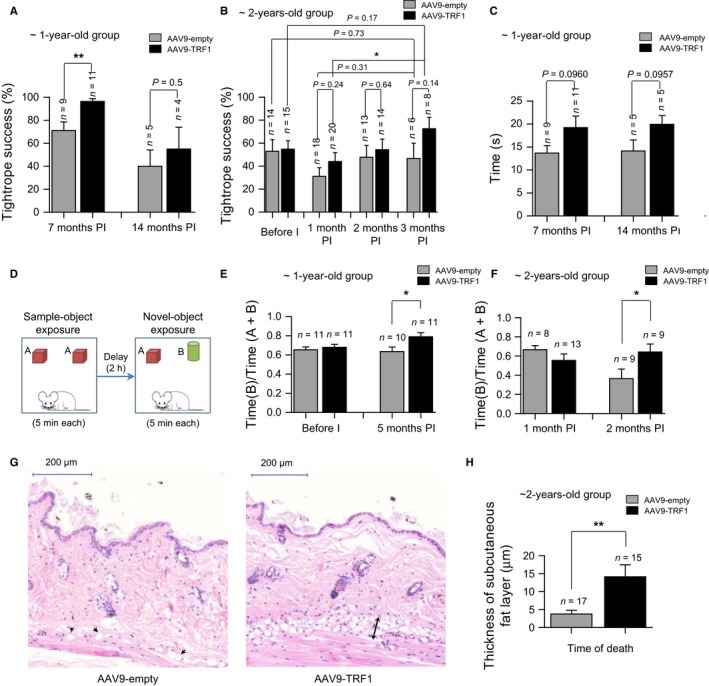
AAV9‐TRF1 gene therapy improves health span in aging mice. (A,B) Neuromuscular coordination using tightrope test in 1‐year‐old (A) and 2‐year‐old (B) mice injected with AAV9‐TRF1 compared to AAV9‐empty vector. (C) Neuromuscular coordination using rotarod test in 1‐year‐old mice. (D) Design of the object recognition test. (E,F) Object recognition test in 1‐year‐old (E) and 2‐year‐old (F) mice. Results show the ratios of time spent investigating the new object vs. the total time spent with both objects. (G) Representative hematoxylin–eosin images of a back skin section of AAV9‐empty and AAV9‐TRF1‐injected mice. In the image of the AAV9‐empty‐injected mouse, black arrows indicate residual adipocytes. In the image of the AAV9‐TRF1‐injected mouse, a black arrow indicates a subcutaneous fat layer. (H) Thickness of the subcutaneous fat layer of mice injected with AAV9‐empty and AAV9‐TRF1 at 2 years old at the time of death. Time points at time postinjection (PI) are indicated. Student's *t‐*test was used for statistical analysis. Error bars represent SEM. *P <* *0.05; **P <* *0.01; *n* indicates the number of mice.

Progressive loss of neuromuscular function is a characteristic of aging (Ingram & Reynolds, 1986). Thus, we first evaluated neuromuscular function in AAV9‐TRF1‐treated mice compared to AAV9‐empty‐treated controls in two sets of experiments: the tightrope test and rotarod test. Mice treated with AAV9‐TRF1 at 1 year of age showed a statistically significant improvement of neuromuscular coordination in the tightrope test at 7 months postinjection compared to controls treated with the empty vector, and this difference was maintained at later time points although the results did not reach statistical significance (Fig. 3A). A similar trend was also observed in the 2‐year‐old group although it did not reach significance (Fig. 3B). In the rotarod test, we also found a trend for improved performance in the 1‐year‐old old group treated with AAV9‐TRF1 compared to the empty‐vector‐treated group, although the differences did not reach significance (Fig. 3C). Thus, AAV9‐TRF1 treatment improved neuromuscular coordination in the 1‐year‐old group, and these differences were lower in the 2‐year‐old group.

#### Cognitive impairment

As AAV9 vectors can cross the blood–brain barrier and we indeed found TRF1 mRNA levels increased in the brain (Fig. 2B), we next investigated the effects of AAV9‐TRF1 gene therapy on cognitive function. To this end, we used the so‐called object recognition test, which is widely used to assess cognitive decline associated with aging (Fig. 3D) (Scali *et al*., 1997). At 5 months post‐treatment with AAV9‐TRF1, 1‐year‐old mice showed improvement in recognition memory scores compared to mice treated with the empty vector (Fig. 3E). We also observed improved cognitive function in the 2‐year‐old mice treated with AAV9‐TRF1 vectors at 2 months postinjection compared to mice treated with the empty vector (Fig. 3F). Thus, AAV9‐TRF1 gene therapy significantly improves memory scores in both the 1‐year‐old and 2‐year‐old groups.

#### Skin aging

Another characteristic of aging is the loss of the subcutaneous fat layer, which increases the risk of skin injury, reduces the ability to maintain body temperature, and raises the probability of infection (Shimokata *et al*., 1989). Thus, we measured the thickness of the subcutaneous fat layer at the time of death of mice in the 2‐year‐old group. Interestingly, mice from the 2‐year‐old group treated with AAV9‐TRF1 showed a significantly thicker fat layer than the empty‐vector‐treated controls at their time of death (Fig. 3G,H), again showing a beneficial effect of AAV9‐TRF1 therapy.

#### Age‐related anemia

**Figure 4:**
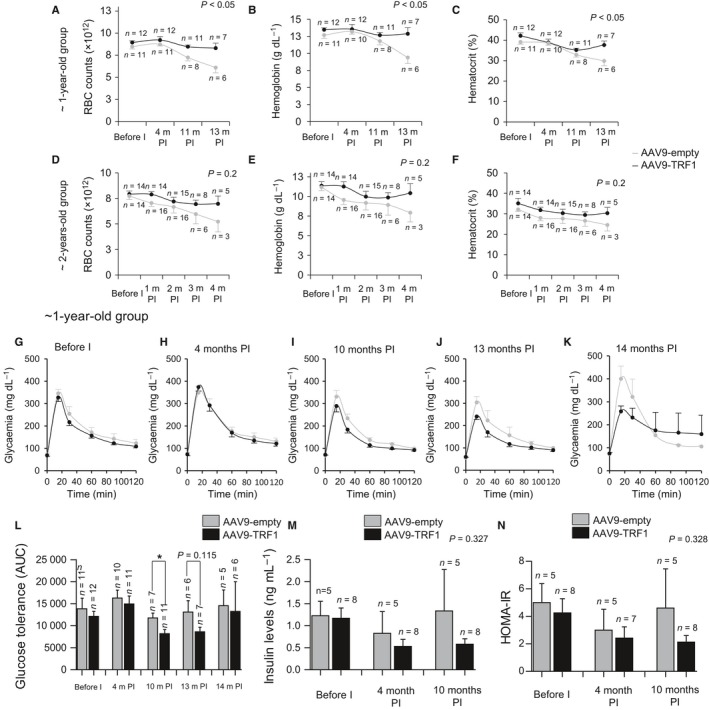
AAV9‐TRF1 gene therapy rescues age‐related anemia condition and delays glucose homeostasis dysregulation. (A–F) Blood analysis for mice injected with AAV9 vectors at 1 year old (A–C) and at 2 years old (D–F). Red blood cell count (RBC) changes (A,D), hemoglobin levels (g/dL) (B,E), and hematocrit percentage (C,F). Time points at time postinjection (PI) are indicated. (G–L) Intraperitoneal glucose tolerance test (IP‐GTT) performed on mice injected with AAV9‐TRF1 and AAV9‐empty at 1 year of age. Glucose curves before injection (G), at 4 months postinjection (H), 10 months postinjection (I), 13 months postinjection (J), 14 months postinjection (K), and AUC (area under the curve) values at these time points (L) are represented. (M) Fasting insulin levels measured in AAV9‐TRF1 mice compared to controls, injected at 1 year of age before injection, at 4 and 10 months postinjection. (N) HOMA‐IR assessment using previous data on IP‐GTT and on fasting insulin levels. Student's *t*‐test was used for statistical analysis. Error bars represent SEM. *P <* *0.05; *n* indicates the number of mice.

Blood chemistry and composition vary with the aging process. Anemia is a common chronic condition appearing with age (Ferrucci & Balducci, 2008; Patel, 2008; Berliner, 2013). Anemia can be caused by various age‐related events occurring in the organism, such as telomere shortening (Herrera *et al*., 1999; Beier *et al*., 2012). Thus, to study the effects of AAV9‐TRF1 treatment on blood changes with age, we followed longitudinally red blood cell (RBC) counts in both the AAV9‐TRF1 and AAV9‐empty‐treated cohorts. To this end, we collected blood at different time points in both groups of mice. As expected, we found a decline in RBCs with age in both 1‐year‐ and 2‐year‐old groups treated with the empty vector (Fig. 4A,D). We also found a decrease in hemoglobin (Hb) and hematocrit (Hct) levels in both age groups (Fig. 4B–C,E–F). Interestingly, mice treated with AAV9‐TRF1 at 1 year of age showed significantly higher RBC counts compared to the control at late time points (Fig. 4A). Analogous improvement was also noticed in the hemoglobin (Fig. 4B) and hematocrit (Fig. 4C) levels. A similar trend was also observed in mice injected at 2 years of age, although differences did not reach statistical significance in this old group (Fig. 4D–F). These observations suggest that AAV9‐TRF1 treatment improves the chronic anemia condition acquired during mouse physiological aging.

#### Glucose Intolerance

Glucose homeostasis likewise becomes dysregulated with age (Bailey & Flatt, 1982). An association of impaired insulin secretion and glucose intolerance with short uncapped telomeres has been established (Kuhlow *et al*., 2010). In particular, short or dysfunctional telomeres trigger a DNA damage response, which can lead to cellular senescence of adult islet beta cells, thus resulting in dysregulation of insulin secretion. Here, we studied whether AAV9‐TRF1 treatment can provide better telomere protection and decrease glucose homeostasis dysregulation associated with physiological aging. To assess glucose tolerance, we performed an intraperitoneal glucose tolerance test (IP‐GTT). We observed a significant improvement in glucose tolerance at 10 months postinjection in the 1‐year‐old AAV9‐TRF1‐treated mice compared to control mice treated with the empty vector (Fig. 4G–L). The same tendency was seen in the fasting insulin level, which is considered to be an indicator of insulin resistance and this level increases with age (Lindberg *et al*., 1997). Levels of fasting insulin showed a lower trend in AAV9‐TRF1‐injected mice compared to controls at late time points postinjection (Fig. 4M). Finally, AAV9‐TRF1‐injected mice also showed a trend of improvement in homeostatic model assessment (HOMA‐IR) compared to control mice treated with the AAV9‐empty vector (Fig. 4N), again suggesting improved insulin sensitivity in the AAV9‐TRF1‐treated cohorts (Heikkinen *et al*., 2007b). Together, these findings suggest that AAV9‐TRF1 gene therapy can improve glucose intolerance associated with age.

To study whether the mice expressing high levels of TRF1 were also the ones showing less age‐related pathologies, we determined the percentage of cells showing high TRF1 expression in muscle. We found that the percentage of cells with high TRF1 expression moderately correlated with improvement in the tightrope test (*R *=* *0.59; Fig. S3A) as well as in the object recognition test (*R *=* *0.51; Fig. S3B). There was also a strong correlation between the % of cells with high TRF1 expression and red blood cell counts (*R *=* *0.98; Fig. S3C) but not with the AUC in the glucose tolerance test (Fig. S3D).

### AAV9‐TRF1 treatment does not affect cancer incidence

**Figure 5:**
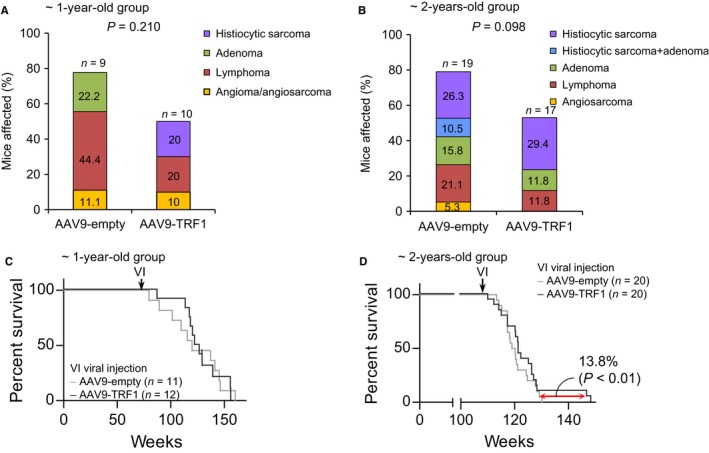
AAV9‐TRF1 treatment does not affect cancer incidence and median lifespan in aging mice. (A,B) Percentage of mice which developed tumors, for mice injected at 1 year (A) and at 2 years (B) of age. (C,D) Survival curves for mice injected with AAV9‐TRF1 compared to AAV9‐empty vectors at 1 year (C) and 2 years (D) old. The determined difference in maximum survival for the 10% longest‐lived mice for mice injected at 2 years of age. Student's *t‐*test was used for statistical analysis. *n* indicates the number of mice.

Increasing age is the highest risk factor for cancer development both in humans and in mice (White *et al*., 2014; Pawelec, 2017). To assess the effects of AAV9‐TRF1 gene therapy on cancer incidence, we performed a full pathological analysis at the time of death in both AAV9‐TRF1‐ and AAV9‐empty‐treated mouse cohorts. Mice treated with AAV9‐TRF1 at 1 and at 2 years of age showed a tendency to show lower cancer incidence compared to the empty‐vector‐treated mice, although the differences did not reach significance (Fig. 5A,B). This illustrates that increased TRF1 expression is not rendering these mice to be tumor prone.

Another indication of absence of deleterious secondary effects associated with increased TRF1 expression is provided by the fact that median survival did not differ between control and AAV9‐TRF1‐treated mice in any of the age groups (Fig. 5C,D). Interestingly, mice injected with AAV9‐TRF1 at 2 years of age showed increased maximum lifespan compared to the controls when comparing the longest‐lived mice in each cohort (Fig. 5D).

### AAV9‐mediated TRF1 overexpression is maintained with age

**Figure 6:**
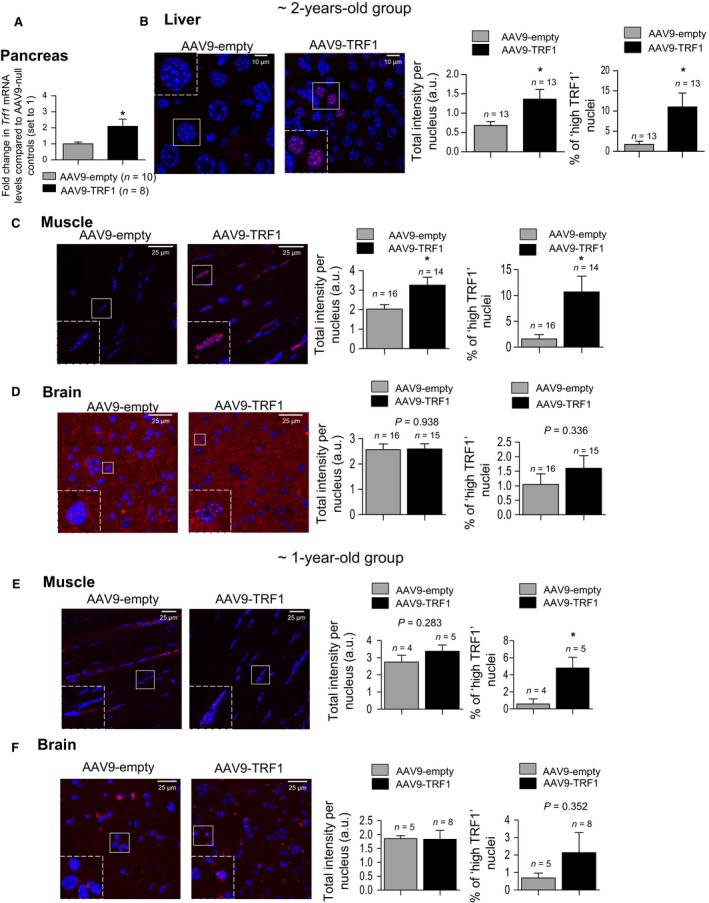
Sustained TRF1 overexpression upon AAV9‐TRF1 treatment in mice. (A) *Trf1* mRNA levels determined by RT–qPCR in pancreatic tissue in mice injected at 2 years of age with AAV9‐TRF1 vector compared to mice injected with AAV9‐empty vector (set to 1). *Trf1* mRNA values are normalized to GAPDH. (B–D) Representative images of TRF1 (in red) and DAPI (in blue), mean TRF1 expression levels and percentage of ‘high TRF1’ nuclei in mice injected with AAV9‐empty and AAV9‐TRF1 vectors at 2 years of age measured at the humane end point in liver (B), muscle (C), and brain (D). (E, F) Representative images of TRF1 (in red) and DAPI (in blue), mean TRF1 expression levels, and percentage of ‘high TRF1’ nuclei in mice injected with AAV9‐empty and AAV9‐TRF1 vectors at 1 year of age, measured at the humane end point in muscle (E) and brain (F). Expression levels are presented as total intensity per nucleus. Student's *t‐*test was used for statistical analysis. Error bars represent SEM. *P <* *0.05; *n* indicates the number of mice.

Previous reports have shown that AAV9 vectors allow for a long‐term expression of the genes that they carry (Bernardes de Jesus *et al*., 2012; Nizzardo *et al*., 2015). To address whether AAV9‐TRF1 treatment allowed for long‐term TRF1 expression, we compared TRF1 mRNA and protein levels at the humane end point in our 2‐year‐old mouse cohort in which all mice had been already sacrificed. We found significantly higher TRF1 mRNA levels in the pancreas of AAV9‐TRF1‐treated mice compared to the controls (Fig. 6A). We also found significantly higher TRF1 protein levels as determined by immunofluorescence in several mouse tissues, such as liver and muscle (Fig. 6B,C). Interestingly, in agreement with the fact that AAV9 vectors are nonintegrative and dilute as the cells divide, we noticed a decreased concentration of AAV9‐TRF1‐targeted cells with aging in all tissues (Figs 2C, and 6E,F), suggesting that even though TRF1 overexpressing cells are present, they are present in lower numbers as the tissues age. For this reason, we set to calculate the percentage of nuclei expressing high TRF1 levels, which reflects the cells maintaining high TRF1 expression at different time points. In most of the tissues analyzed, we identified significantly higher abundance of nuclei with ‘high TRF1’ levels in AAV9‐TRF1‐injected mice compared to the controls (Fig. 6B–F). Thus, in muscle tissue of mice injected at 1 year of age, we could not detect any difference comparing mean TRF1 levels between the AAV9‐TRF1‐treated mice and the empty‐vector controls. However, we readily detected a highly significant increase in ‘high TRF1’ nuclei in the AAV9‐TRF1‐treated mice compared to the controls, confirming that at late time points, TRF1 is still overexpressed (Fig. 6E), however, in fewer cells than at earlier time points (Fig. 2D).

### AAV9‐TRF1 gene therapy does not have negative effects on telomere length and provides protection from the accumulation of short telomeres

**Figure 7:**
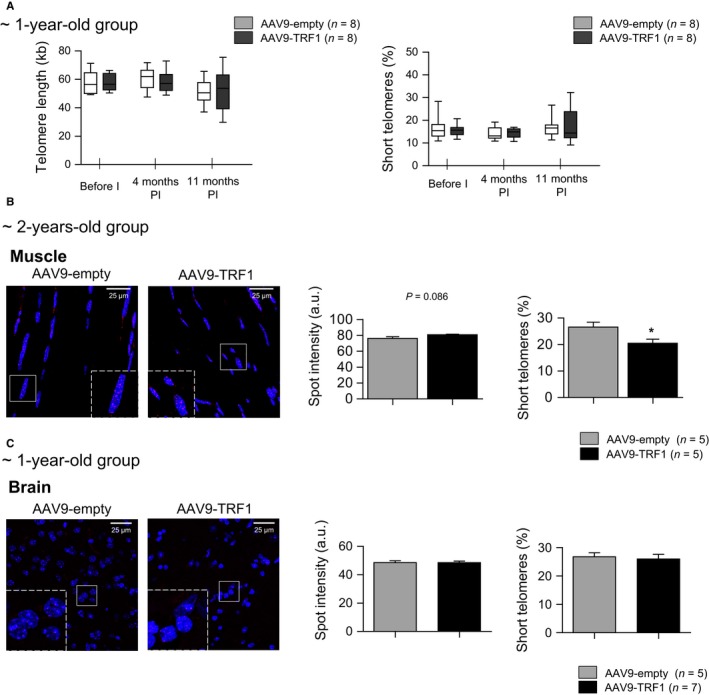
AAV9‐TRF1 gene therapy has no effect on mean telomere length and rescues accumulation of short telomeres. (A) Characterization of telomere length measured by high‐throughput Q‐FISH (HT Q‐FISH). Comparison of mean telomere length and percentage of short telomeres measured in peripheral white blood cells in mice injected at 1 year of age with AAV9‐TRF1 vector compared to the control. (B,C) Representative images of telomere probe Cy3 (in red) and DAPI (in blue) and quantification of telomere length and percentage of short telomeres measured in muscle in mice injected with virus vectors at 2 years of age (B) and in brain of mice injected at 1 year of age (C). Analysis performed at the time of death. Student's *t‐*test was used for statistical analysis. Error bars represent SEM. *P <* *0.05; *n* indicates the number of mice.

Next, we set to address the long‐term effects of AAV9‐TRF1 gene therapy on telomere length. We first performed a longitudinal study on telomere length dynamics in blood peripheral leukocytes using the high‐throughput Q‐FISH (HT Q‐FISH) technique. We did not observe any significant difference in the mean telomere length or percentage of short telomeres in white blood cells or tissues between 1‐year‐old injected experimental mice and the control groups (Fig. 7A, Fig. S4). Q‐FISH performed on muscle tissue of mice injected at 2 years of age and in the brain of mice injected at 1 year of age also did not show any difference in the mean telomere length (Fig. 7B,C). Interestingly, the percentage of short telomeres, determined here within a control group as telomeres with intensities lower than the 25th percentile, was lower in muscle tissue of mice injected with AAV9‐TRF1 compared to controls (Fig. 7B). These results highlight that TRF1 overexpression through AAV9‐TRF1 gene therapy does not trigger XPF‐mediated telomere shortening, as it was observed in the case of constitutive TRF1 overexpression in a transgenic mouse model (Muñoz *et al*., 2009). Moreover, TRF1 overexpression in muscle tissue led to better protection of telomeres, delaying the accumulation of the short telomeres.

### AAV9‐TRF1 increases telomere protection in the hippocampus area of the brain of mice

Based on the finding that AAV9‐TRF1 gene therapy was able to delay the accumulation of short telomeres, we further addressed the question of whether it also prevents DNA damage at dysfunctional telomeres. As an improvement in recognition memory was observed, the hippocampus was further investigated. The hippocampus is a brain structure that plays a central role in the formation of new memories (Cohen & Eichenbaum, 1993). The hippocampus exhibits neurogenesis, which continues through adulthood in a substructure named the dentate gyrus (Gross, 2000). Aging and other conditions affect processes of neurogenesis, and this leads to impairment of cognitive function (Drapeau *et al*., 2003; Jin *et al*., 2003). As TRF1 is important for telomere protection and moreover plays a role in stem cell function, we proposed that its deficiency may have molecular consequences and affect the stem cell pool of the dentate gyrus, thus leading to impaired neurogenesis and memory with age. In this case, AAV9‐TRF1 gene therapy may rescue this impairment to some extent.

**Figure 8:**
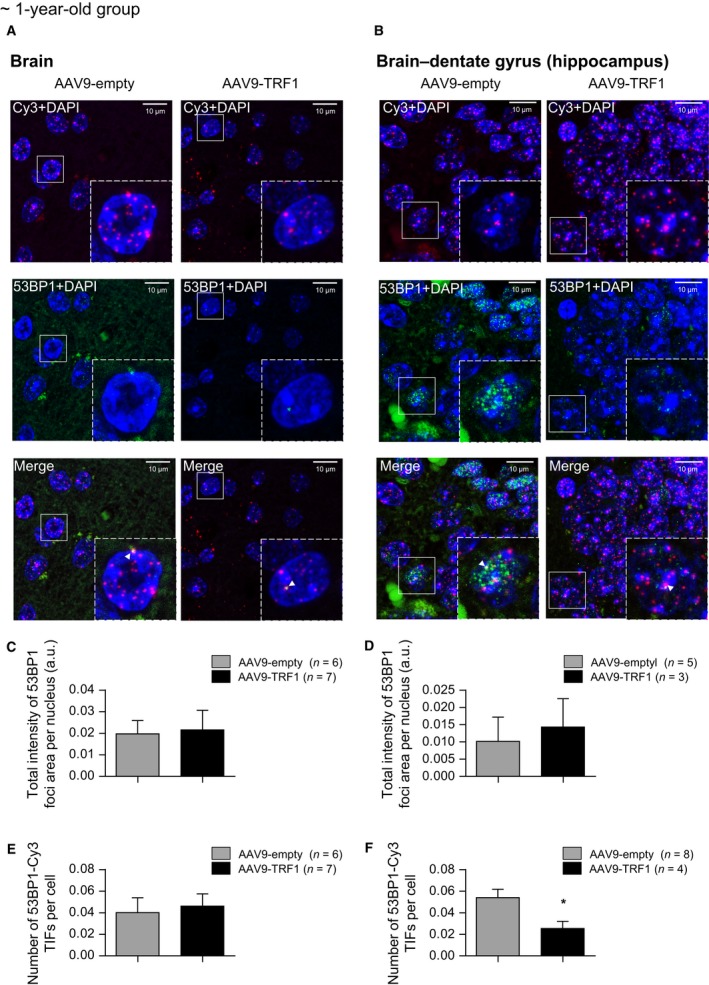
AAV9‐TRF1 gene therapy reduces DNA damage levels at telomeres in the brain. (A,B) Representative images for immuno‐FISH analysis on DNA damage and telomere‐associated DNA damage in the whole brain (A) and specifically in the dentate gyrus of the hippocampus (B). Cy3 probe is presented in red, 53BP1 in green, and DAPI in blue. The representative images are merged from the Z‐stack of images. Colocalization events (white arrows) were only counted as positive when detected in the individual confocal images. (C,D) Analysis of total DNA damage determined as the total intensity of 53BP1 foci per nucleus in the whole brain (C) and in the dentate gyrus area (D). (E,F) Analysis of telomere‐induced DNA damages determined as the number of Cy3‐53BP1 colocalization events per cells in the whole brain (E) and the dentate gyrus zone (F). Analysis performed at the time of death. Student's *t*‐test was used for statistical analysis. Error bars represent SEM. *P <* *0.05; *n* indicates the number of mice.

We first compared the DNA damage status in the brain of mice injected with AAV9‐TRF1 and control vector at the adult age. Total DNA damage was determined as area covered by 53BP1 foci. We did not observe any difference in total DNA damage in the whole brain, neither in the dentate gyrus area or the hippocampus (Fig. 8A–D). Next, DNA damage at telomeres was determined by immuno‐FISH analysis as the number of TIFs (Telomere Dysfunction Induced Foci)—spots of colocalization of 53BP1 foci and telomere probe Cy3 foci. Although we did not see differences in whole‐brain sections (Fig. 8A,E), we observed significantly less TIFs in the dentate gyrus of mice treated with AAV9‐TRF1 gene therapy compared to the empty‐vector group (Fig. 8B,F). Thus, AAV9‐TRF1 gene therapy seems to prevent DNA damage at telomeres in the dentate gyrus, which may have led to the improvement of the memory function observed in the object recognition test (Fig. 3E,F).

## Discussion

TRF1 is a central component of the shelterin complex, with an important role in telomere protection (Smith & de Lange, 1997; Palm & de Lange, 2008; Diotti & Loayza, 2011). TRF1 also marks and is essential for both adult stem cells and pluripotent cells (Schneider *et al*., 2013). Interestingly, TRF1 levels decrease with increasing cell passages *in vitro* (Hohensinner *et al*., 2016), suggesting that TRF1 levels may also decrease with organismal aging.

Here, we make the unprecedented finding that TRF1 levels decrease during mouse and human aging *in vivo*. This poses the interesting hypothesis that reduced TRF1 levels with aging may lead to telomere uncapping and telomere‐induced DNA damage (Martínez *et al*., 2009; Sfeir *et al*., 2009), as well as to loss of tissue homeostasis (Schneider *et al*., 2013), eventually leading to tissue dysfunction and organismal aging. To test this hypothesis, here we set to rescue TRF1 levels in aging by overexpressing TRF1 in adult and old mice. We previously described that constitutive transgenic overexpression of TRF1 results in telomere shortening *in vivo* (Muñoz *et al*., 2009). To overcome this problem, and to achieve a transient overexpression of TRF1, we used adeno‐associated gene therapy vectors. As recombinant AAV9 vectors are nonintegrative and can transduce the majority of mouse tissues, they provide transient overexpression of the target protein in several mouse tissues (Ayuso *et al*., 2010).

We describe here that mice treated with AAV9‐TRF1 gene therapy showed significant improvements in health span, such as delayed anemia, and improvements in neuromuscular coordination, memory, epithelial barrier fitness, and glucose metabolism. Importantly, overexpression of TRF1 using AAV9 vectors did not trigger telomere shortening.

The AAV9‐TRF1 therapy affected TRF1 levels in postmitotic tissues, and this may contribute to the effect of TRF1 gene therapy on aging. Although many of the cells in postmitotic tissues do not proliferate, these tissues also contain stem cell compartments, which are responsible for maintaining tissue homeostasis throughout life. This is the case in the brain, the heart, and the muscle. For example, the brain exhibits neurogenesis, which continues throughout adulthood (Gross, 2000). Importantly, we have previously demonstrated that stem cell compartments exhibit increased TRF1 expression and that TRF1 is necessary for tissue homeostasis even in the presence of a normal telomere length (Martínez *et al*., 2009; Schneider *et al*., 2013). Thus, TRF1 loss even in the presence of long telomeres can trigger severe defects in tissue homeostasis. Note that our group has previously demonstrated through whole‐genome ChIP sequencing that TRF1 binding is restricted to telomeres and this is not influenced by shorter telomeres owing to telomerase deficiency (Garrobo *et al*., 2014). Therefore, it is unlikely that transient increased TRF1 expression owing to AAV9‐TRF1 gene therapy may have major effects on gene expression elsewhere in the genome. This is in contrast to other shelterin components like RAP1, which can affect gene expression and this effect is influenced by telomere length (Martinez *et al*., 2010; Ye *et al*., 2014). We show here that TRF1 levels decrease with mouse aging in both highly proliferative tissues such as the skin and intestine, as well as in some postmitotic tissues such as muscle. As the deficiency of TRF1 leads to impaired telomere protection, this loss may play a role in age‐associated impairments in the stem cell compartments as well. In addition, other processes associated with aging such defective DNA repair mechanisms could also result in deprotected telomeres (Samper *et al*., 2000; Goytisolo *et al*., 2001; Espejel *et al*., 2004a; Espejel *et al*., 2004b; Tarsounas *et al*., 2004; Grach, 2013).

Interestingly, we observed less telomere‐associated DNA damage foci upon gene therapy in the hippocampus area of the brain. Previous reports showed that age‐associated accumulation of DNA damage in the hippocampus leads to impaired neurogenesis (Lemaire *et al*., 2000) and causes age‐related cognitive alterations such as impaired memory function (Drapeau *et al*., 2003). In this regard, the improved memory scores observed here upon AAV9‐TRF1 treatment may suggest that AAV9‐TRF1 gene therapy can delay age‐related decline in hippocampal neurogenesis (Lemaire *et al*., 2000; Drapeau *et al*., 2003; Nacher *et al*., 2003), thus improving memory performance.

The effect of TRF1 on telomeres appears to be influenced by the level of TRF1 expression. In a previous publication (Muñoz *et al*., 2009), we highly overexpress TRF1 in a constitutive manner over the lifetime of mice. This persistent TRF1 overexpression leads to telomere shortening mediated by the XPF nuclease. However, in this work, TRF1 is transiently expressed through AAV9‐TRF1 gene therapy. After a single injection of these nonintegrative vectors, the TRF1 signal becomes diluted with time in replicative tissues. Therefore, the levels of TRF1 overexpression achieved are much lower. As demonstrated here, the levels of TRF1 overexpression needed to negatively regulate telomere length are not reached after the TRF1 gene therapy as telomere shortening was not observed. Indeed, we observed fewer short telomeres and telomere‐associated DNA damage in some tissues, which is consistent with *in vitro* data showing beneficial effects of TRF1 overexpression in aged cells (Hohensinner *et al*., 2016). Regarding, how TRF1 stabilizes telomeres, we have previously demonstrated that persistent TRF1 downregulation in bone marrow stem cells can lead to cellular senescence of stem cells and to a compensatory hyperproliferation of the remaining stem cells to maintain tissue homeostasis. This compensatory hyperproliferation also leads to telomere shortening (Beier *et al*., 2012). Thus, decreased TRF1 levels with aging may also lead to loss of stem cells and the extra proliferation of the remaining progenitor cells, thus leading to telomere loss. Increased TRF1 expression may attenuate this.

In conclusion, we describe here an age‐related loss of one of the key elements of telomere protection, TRF1. We further demonstrate that TRF1 decreased levels contribute to the aging phenotype, at least partially. In particular, transient and moderate TRF1 overexpression using recombinant adeno‐associated viral vectors leads to beneficial effects both on molecular and physiological levels, ensuring better telomere protection and a prolonged health span.

## Materials and Methods

### Mice and animal procedures

Experiments were performed with wild‐type mice of a > 95% C57BL/6 background. All of the data were collected in a quantitative way, and subjective evaluations were not made. Mice were produced and housed in the specific‐pathogen‐free animal house of CNIO, Madrid. All procedures performed on mice were approved by the CNIO‐ISCIII Ethics Committee for Research and Animal Welfare (CEIyBA). Mice were treated according to Spanish laws and the guidelines of the Federation of European Laboratory Animal Science Associations (FELASA).

Mice were tail‐vein‐injected with a dose of 2 × 10^12^ viral genomes per mouse–AAV9‐TRF1 in the experimental group and AAV9‐empty in the control group. Injections were performed at different ages: adult mice of ~ 1 year of age (64–65 weeks old) and old mice ~ 2 years of age (109–110 weeks old). Between different groups within the same age, an equal ratio of males/females was maintained.

### Recombinant AAV9 vectors

Mouse TRF1 was cloned into *pBABE puro* plasmid vector at CNIO (Madrid), and AAV9 containing TRF1 and control vectors were produced and purified as described previously (Matsushita *et al*., 1998; Ayuso *et al*., 2010). Briefly, recombinant AAV9 vectors were produced through triple transfection of HEK293 cells by plasmid carrying a TRF1 expression cassette, plasmid carrying the AAV *rep* and *cap* genes, and an adenovirus helper plasmid. AAV9 vector purification was achieved by CsCl gradient centrifugation, following CsCl removal by dialysis against PBS and filtration. Titers of viral particles were determined by quantitative RT–PCR.

### RNA isolation and RT–qPCR

Analyzed tissues were homogenized, and total RNA was isolated using the RNeasy Mini Kit (QIAGEN, Venlo, Limburg, Netherlands). The concentration and purity of the RNA were determined with a NanoDrop ND‐1000 spectrophotometer. Total RNA was retrotranscribed using the iScript cDNA Synthesis Kit (Bio‐Rad, Hercules, California, USA), according to the manufacturer's guidelines. Expression levels of Trf1 mRNA were determined by real‐time PCR, performed using Power SYBR Green PCR Master Mix (Applied Biosystems, Waltham, Massachusetts, USA) in an ABI 7900HT Fast Real‐Time PRC System (Applied Biosystems). Each reaction was performed in triplicate and normalized to GAPDH mRNA levels as an endogenous control. Sequences of the mouse primers used for quantitative real‐time PCR in this work are listed as follows: *TRF1*‐Fw: 5′‐TCT AAG GAT AGG CCA GAT GCC A‐3′, *TRF1*‐Rv: 5′‐CTG AAA TCT GAT GGA GCA CGT C‐3′, *GAPDH*‐Fw: 5′‐TTC ACC ACC ATG GAG AAG GC‐3′, *GAPDH*‐Rv: 5′‐CCC TTT TGG CTC CAC CCT‐3′.

### Immunofluorescence analysis

Immunofluorescence analysis was performed on paraffin‐embedded tissue sections as described previously (Martínez *et al*., 2009; Tejera *et al*., 2010). To target TRF1, a homemade rat monoclonal anti‐TRF1 antibody and polyclonal goat anti‐rat Alexa 555 as a secondary antibody were used. Images were captured on a confocal ultraspectral microscope Leica TCS‐SP5‐WLL. Fluorescence intensities were analyzed using Definiens Developer Cell software.

### Blood samples and cell counts

Blood samples were taken at different time points over the lifespan by facial vein puncture (~ 50 μL) and collected in EDTA anticoagulant tubes. Blood cell counts, hematocrit (Hct), and hemoglobin (Hb) concentrations were measured on an *Abacus Junior* Vet veterinary hematology analyzer.

### High‐throughput Q‐FISH (HT Q‐FISH)

To perform a longitudinal study on telomere length dynamics in mouse peripheral white blood cells, blood samples were collected as described in the section above at the indicated time points after AAV9 injection. Blood samples were processed with erythrocyte lysis using Buffer EL (QIAGEN) and frozen in 10% DMSO/FBS. Prior to proceeding with the protocol, blood samples were defrozen and plated in poly‐L‐lysine precoated clear bottom black‐walled 96‐well plates (Greiner, Kremsmünster, Upper Austria, Austria). Samples were analyzed in duplicate. The HT Q‐FISH protocol was performed as described in Canela *et al*. (2007). To convert telomeres fluorescence values into kb, we used standard cell lines with stable telomere length: L5178Y‐R (79.7 kb), HeLa1211 (23.8 kb), and CCRF‐*CEM* (7.5 kb). Images were acquired on an Opera High Content Screening System (PerkinElmer, Inc., Waltham, Massachusetts, USA) and analyzed with Acapella Image analysis software (PerkinElmer, Inc.).

### Telomere Q‐FISH analysis

Telomere analysis was performed on paraffin‐embedded tissue sections, which were deparaffinized, hybridized with a PNA‐telomeric probe, and treated as described in Zijlmans *et al*. (1997). Images were captured on the confocal ultraspectral microscope Leica TCS‐SP5‐WLL. Analysis of images was performed using the Definiens Developer Cell software.

### Immuno‐FISH analysis

To assess the level of DNA damage at telomeres, we performed Immuno‐FISH analysis. First, we performed a Q‐FISH protocol excluding a pepsin digestion step, followed by an immunofluorescence protocol, using rabbit monoclonal anti‐53PB1 antibodies for the primary antibody and polyclonal goat anti‐rabbit Alexa 488 antibodies for the secondary antibody. Images were acquired on a confocal ultraspectral microscope Leica TCS‐SP5‐WLL. Analysis of images was performed using the Definiens Developer Cell software.

### Neuromuscular coordination

Neuromuscular coordination and balance were evaluated in tightrope and rotarod tests. In the tightrope test, mice were placed onto a bar (100 cm long and 1.5 cm diameter). If the mouse was able to remain on the bar for 1 min, it was considered as a success. Percentage of success of five trials was determined. In the rotarod test, mice were placed onto a rod which was rotating with accelerating speed from 4 to 40 rpm during 1 min. The mean time of the latency before falling was measured in three trials.

### Memory analysis

Recognition memory was studied in the object recognition test as described in Bevins & Besheer (2006). Mice were placed in a box to investigate two equal objects for 5 min. In a 2‐h time gap, one of the two objects was replaced with an object of different form and structure, and the mouse was placed back into the box for 5 more min. The memory score was quantified as the time spent investigating a novel object divided by the total time of investigation of both objects.

### Subcutaneous fat thickness

Thickness of the subcutaneous fat layer was measured as described previously (Moynihan *et al*., 2005). Briefly, a total of 20 measurements were performed on three back sections of the skin for each mouse sacrificed at the humane end point. For these measurements, 17 AAV9‐empty and 15 AAV9‐TRF1 mice were used. Measurements were made using Panoramic Viewer software.

### Intraperitoneal glucose tolerance test (IP‐GTT)

To measure the clearance of injected glucose into peripheral tissues, an IP‐GTT test was performed as described (Moynihan *et al*., 2005; Tomás‐Loba *et al*., 2008). Briefly, mice were fasted for 14 h and then injected intraperitoneally with 50% D‐(+)‐glucose solution (2 g/kg body weight). Blood was collected from the tail vein at 0, 15, 30, 60, 90, and 120 min after injection, and blood glucose levels were measured with a glucometer and Glucocard Memory Strips (Arkray Factory, Inc., Japan). Glucose curves on changing of glucose levels with time were represented, and area under the curve (AUC) was calculated.

### Fasting insulin levels and homeostatic model assessment of insulin resistance (HOMA‐IR)

Serum was taken from mice after 14 h of fasting. Insulin levels were measured in blood serum with an Ultra‐Sensitive Mouse Insulin ELISA Kit (Crystal Chem Inc., Downers Grove, IL, USA), following the manufacturer's protocol.

HOMA‐IR was performed as described (Heikkinen *et al*., 2007). HOMA‐IR index was calculated according to the formula:

$$\text{HOMA}-\text{IR}=\frac{\text{FPI}\times \text{FPG}}{22.5\times 18\times 45.5}$$

where FPI is the fasting serum insulin (ng/mL) determined by ELISA, and FPG is the fasting blood glucose concentration (mg/dL).

## Funding

AAV vector generation and production were funded by Ministerio de Economía y Competitividad, Plan Nacional I+D+I (SAF2014‐54886‐R), Spain. Research in Maria Blasco's laboratory is funded by the Ministerio de Economía y Competitividad, Plan Nacional I+D+I (SAF2013‐45111‐R), and by the Fundación Botín.

## Conflict of interest

None declared.

## Supporting information


**Fig. S1.** Changes of TRF1 levels with aging in mice.
**Fig. S2.** RT‐qPCR of shelterin proteins in muscle tissue.
**Fig. S3.** Correlation between percentage of nuclei overexpressing high TRF1 levels and readouts of aging.
**Fig. S4.** AAV9‐TRF1 gene therapy has no effect on mean telomere length.
