# Supplementary material for: Gene therapy with the TRF1 telomere gene rescues decreased TRF1 levels with aging and prolongs mouse health span
Source: Aging Cell. 2017 Sep 24;16(6):1353–68. doi: 10.1111/acel.12677 (PMC5676056; doi:10.1111/acel.12677)
Supplement: Supplementary file 1 — Fig. S1. Changes of TRF1 levels with aging in mice. Fig. S2. RT‐qPCR of shelterin proteins in muscle tissue. Fig. S3. Correlation between percentage of nuclei overexpressing high TRF1 levels and readouts of aging. Fig. S4. AAV9‐TRF1 gene therapy has no effect on mean telomere length. [file ACEL-16-1353-s001.ppt]

## Slide 1
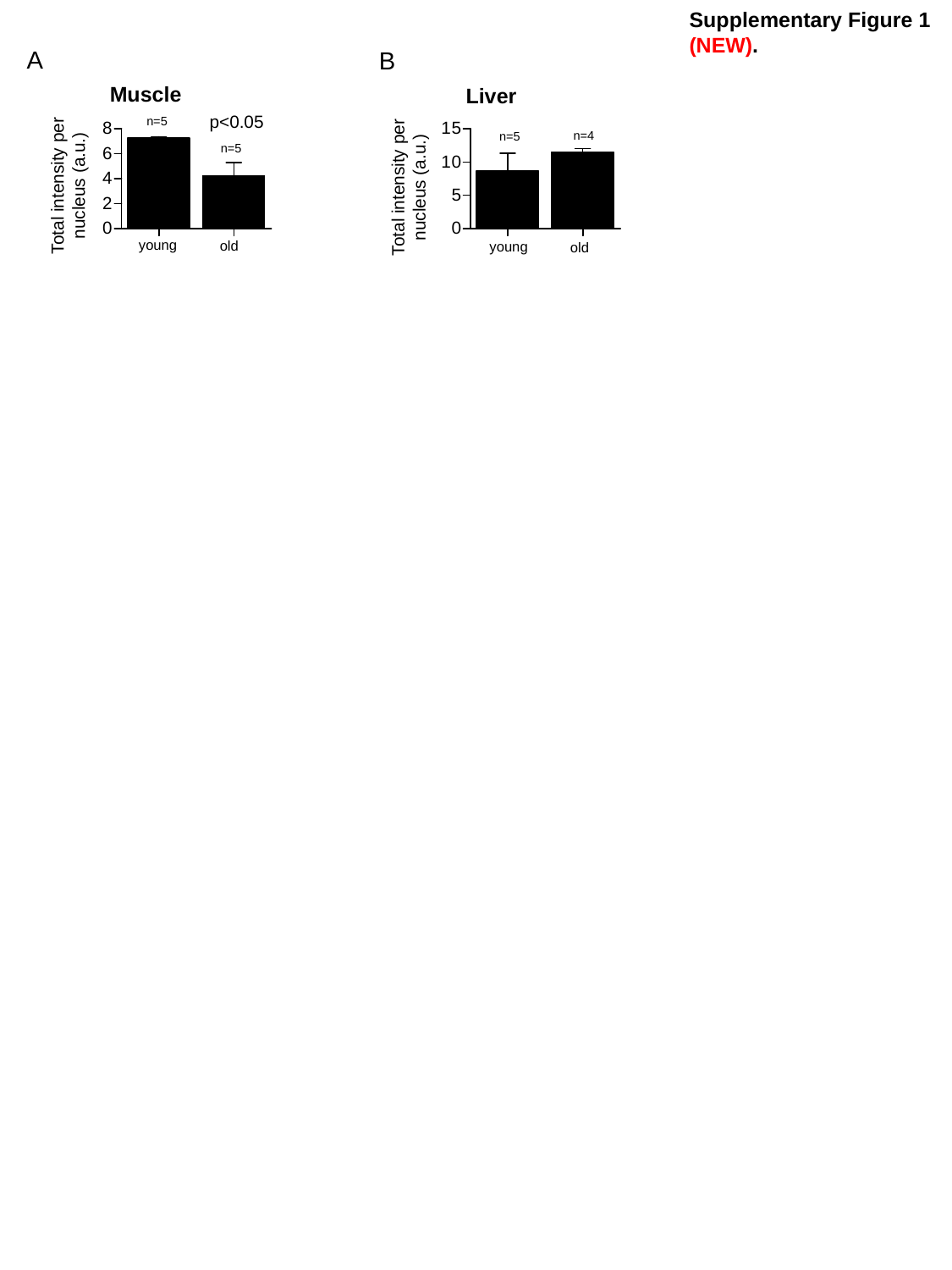

Supplementary Figure 1 (NEW).
A
B
Muscle
Liver
p<0.05
n=5
n=4
n=5
n=5
Total intensity per nucleus (a.u.)
Total intensity per nucleus (a.u.)
young
old
young
old

## Slide 2
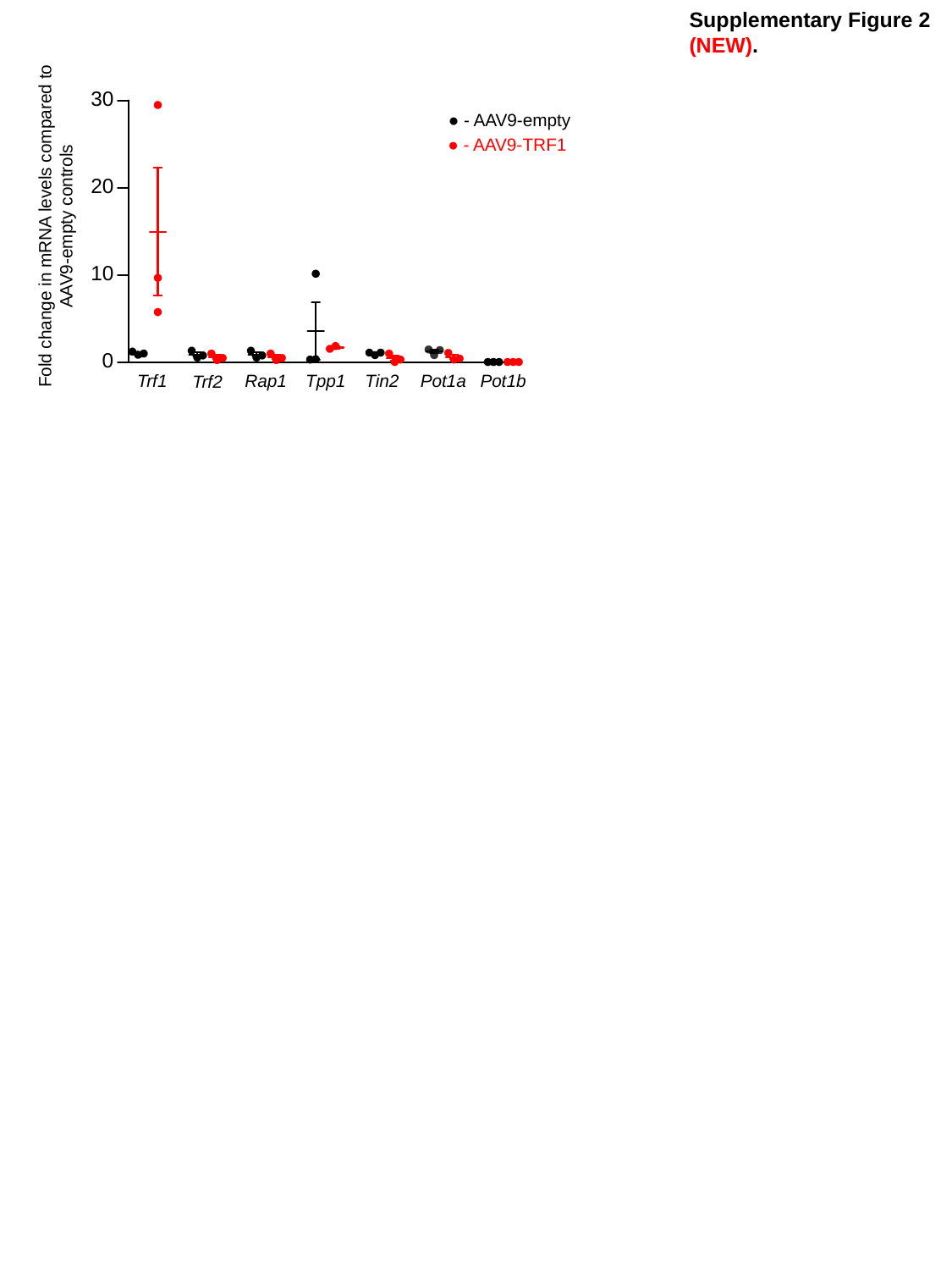

Supplementary Figure 2 (NEW).
● - AAV9-empty
● - AAV9-TRF1
Fold change in mRNA levels compared to AAV9-empty controls
Rap1
Tpp1
Tin2
Pot1a
Pot1b
Trf1
Trf2

## Slide 3
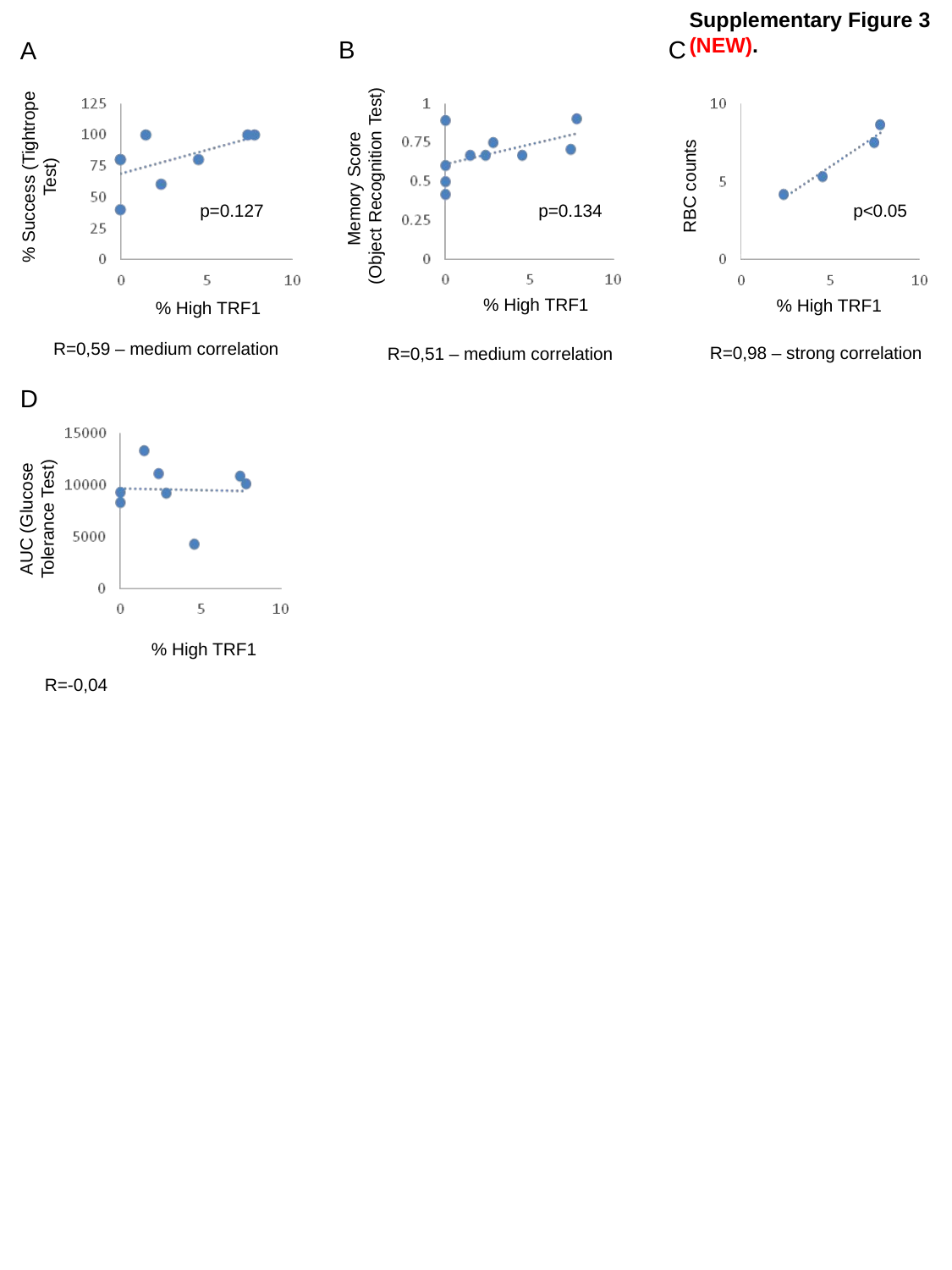

Supplementary Figure 3 (NEW).
B
C
A
% Success (Tightrope Test)
Memory Score
(Object Recognition Test)
RBC counts
p<0.05
p=0.127
p=0.134
% High TRF1
% High TRF1
% High TRF1
R=0,59 – medium correlation
R=0,98 – strong correlation
R=0,51 – medium correlation
D
AUC (Glucose Tolerance Test)
% High TRF1
R=-0,04

## Slide 4
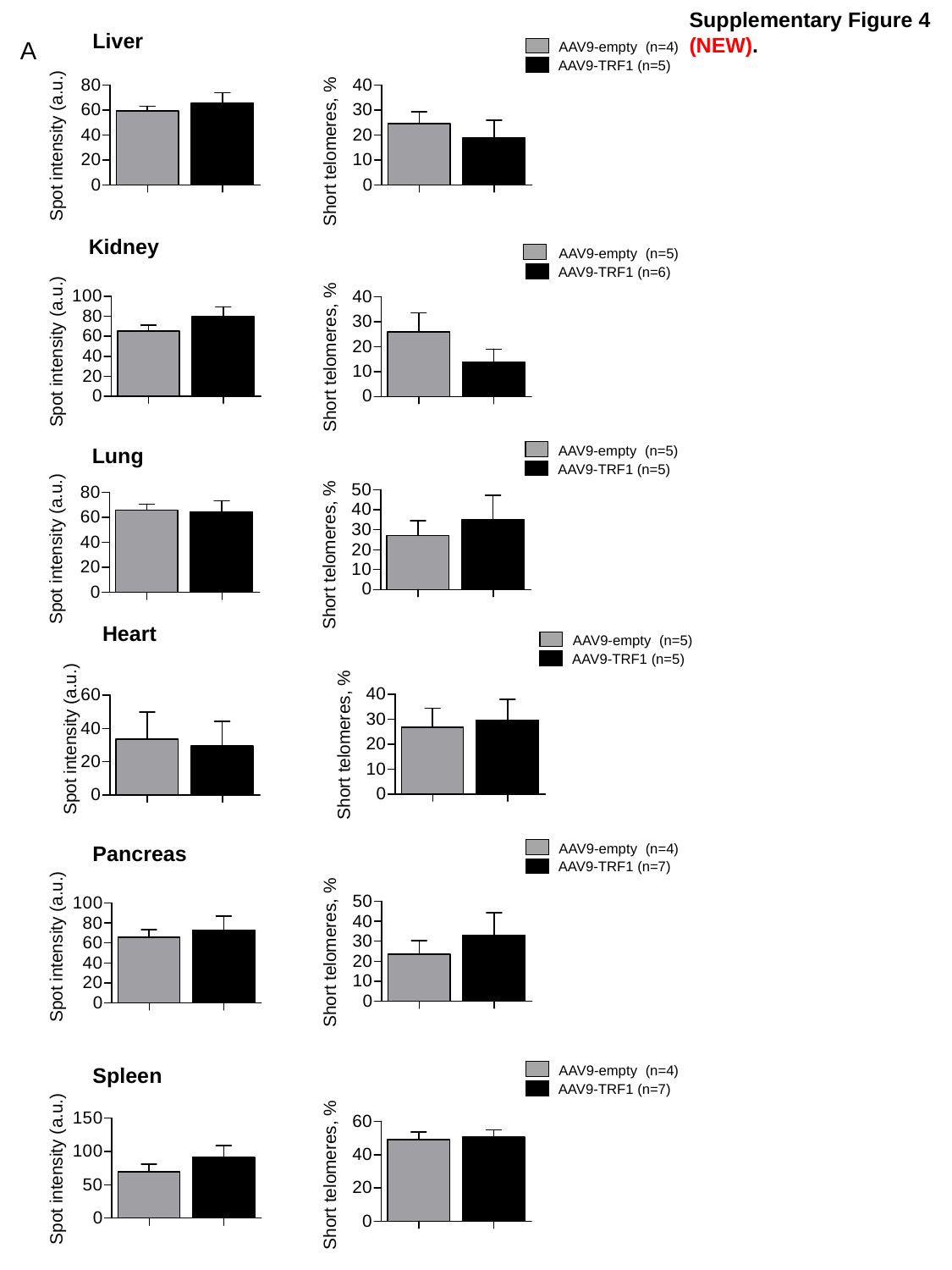

Supplementary Figure 4 (NEW).
Liver
A
AAV9-empty (n=4)
AAV9-TRF1 (n=5)
Spot intensity (a.u.)
Short telomeres, %
Kidney
AAV9-empty (n=5)
AAV9-TRF1 (n=6)
Spot intensity (a.u.)
Short telomeres, %
AAV9-empty (n=5)
Lung
AAV9-TRF1 (n=5)
Spot intensity (a.u.)
Short telomeres, %
Heart
AAV9-empty (n=5)
AAV9-TRF1 (n=5)
Spot intensity (a.u.)
Short telomeres, %
AAV9-empty (n=4)
Pancreas
AAV9-TRF1 (n=7)
Spot intensity (a.u.)
Short telomeres, %
AAV9-empty (n=4)
Spleen
AAV9-TRF1 (n=7)
Spot intensity (a.u.)
Short telomeres, %
